# The effect of designing a rotational planning target volume on sparing pharyngeal constrictor muscles in patients with oropharyngeal cancer

**DOI:** 10.1002/acm2.13052

**Published:** 2020-10-19

**Authors:** Mona Arbab, Huisi Ai, Gregory Bartlett, Benjamin Dawson, Mark Langer

**Affiliations:** ^1^ Department of Radiation Oncology Indiana University School of Medicine Indianapolis IN USA

**Keywords:** oropharyngeal cancer, pharyngeal constrictor muscle, rotational PTV

## Abstract

**Background:**

Planning target volume (PTV) has been used to account for variations in tissue, patient and beam position. In oropharyngeal cancers, an isotropic expanded PTV has been used.

**Aim:**

The aim of this study was to design a new margin formula that would cover the space occupied by an oropharyngeal clinical target volume (CTV) with ±5‐degree rotation around the spine in order to reduce the pharyngeal constrictors overlap with PTV compared to an isotropic expanded PTV.

**Methods:**

We retrospectively evaluated 20 volumetric‐modulated arc therapy (VMAT) plans. In order to perform an off‐axis rotation, a hypothetical point was placed through the center of the cervical spinal canal and the image was then rotated around the longitudinal axis ±5 degrees. This created a new set of CTVs that were combined to form the new rotational PTV. The overlap between the pharyngeal constrictor muscles (PCMs) and both PTVs was then evaluated.

**Results:**

The new rotational PTV causes reduction in the superior PCM overlap in the base of tongue (BOT) lesions compared to tonsillar lesion, 57.8% vs 25.8%, *P* = 0.01, as well as middle PCM overlap, 73% vs 49%, *P* = 0.04. Average percent change for PTV volume and overlap with the superior, middle, and inferior PCMs are as followed: −19%, −37%, −59.4%, and −45.2. The smallest isotropic expansion that covers the new rotational PTV was between 3 and 5mm with the average tumor center shift of 0.49 cm.

**Conclusion:**

This new rotational PTV causes significant reduction of the overlap volume between PCMs and PTVs in order to spare the PCMs compared to isotropic expanded PTV.

## 1 INTRODUCTION

Intensity‐modulated radiation therapy (IMRT) and rotational intensity‐modulated techniques, including volumetric‐modulated arc therapy (VMAT) have been used in patients with head and neck cancer. These techniques offer dose distributions conformal to the tumor with superior sparing of the organs at risk (OARs).^1^ In order not to miss the target, safety margins are applied which account for anatomic motion, delineation errors, and setup errors.^2^ This expanded safety volume is known as planning target volume (PTV). Historically, a uniform margin around the clinical target volume (CTV) has been used to define the PTV. Target displacement can be decomposed into translations and rotations. The rotational component is of great importance especially when the target has a non‐spherical shape or the rotation is off axis, as a small rotation can cause deviation of the dose distribution.^3^, ^4^ As shown in a study by Peng et al, in large targets with irregular shapes, target coverage can decrease significantly when rotational error of 5 degrees or more is present.^5^ Both translations and rotations should be considered to form the swept space of the target in designing precise PTVs, but rotations are typically neglected. In addition, estimating the swept volume of an object with both rotation and translation is not simply solved.^6^, ^7^ Based on a study done by Hong et al considering 20 institutes, the average recommended PTV expansion from CTV was 4.11 mm with a standard deviation of 3.19 mm.^8^. Another study done by Djordjevic et al showed considerable local residual setup error even with daily imaging in head and neck cancers and required PTV margins ranging 4.5 to 9.3 mm for each subregion. This study suggested designing a variable margin related to the tumor site to account for minor cervical deformations.^9^ Most planning systems allow margins to be specified along the “three Cartesian dimensions only,” a limitation attested in the ICRU 83 report.^10^

Based on the American Cancer Society (ACR) reports, the 5‐yr relative survival rates for cancers of the oral cavity and pharynx is 65% and can be as high as 84% in patients with early stage disease.^7^ This emphasizes the importance of reducing tissue morbidity by delivering a more focused radiation treatment. Dysphagia is a potentially a devastating toxicity of radiation therapy (RT) in this population as 59% of head and neck patients report persistent dysphagia at an average of 33‐month follow‐up.^11^ This can lead to limited oral intake and possibility of developing aspirations which can lead to life‐threatening aspiration pneumonia as well as feeding tube dependence. 60% of patients are feeding tube dependent during their treatment course.^12^, ^13^ The cricopharyngeus muscle, inlet of esophagus, superior, middle and inferior constrictors play an important role in the swallowing process.^14^ There are several studies that have evaluated the correlation between dose to pharyngeal constrictors and dysphagia rates. In one study, for a median dose of 50 Gy to superior and middle pharyngeal constrictors, the probability of developing grade 3 and 4 dysphagia is approximately 20% and if the dose is reduced to 22 Gy, the probability of developing dysphagia is as low as 2%.^14^ Another study evaluated the effect of reducing PTV margin on radiation induced toxicity. A 2 mm reduction in the in the PTV margin resulted in a significant reduction of acute dysphagia defined as feeding tube dependence by 50% and late dysphagia from 22% to 11%.^15^ This emphasizes the importance of margin construction based on real anatomic motion rather than uniform expansion of the tumor. Cervical spinal rotation can happen during treatment. In a study done by Kapanen et al, improvement in the formation of thermoplastic masks by making them tighter and improved image matching to vertebrae reduced residual random errors especially by reducing the rotation of vertebrae and head.^16^ The positional uncertainty contributed by rotational displacements are significant. Rotations greater than 3 degrees have been observed.^17^ In a study done by Nakata et al, the setup and rotational shifts in head and neck cancer patients undergoing IMRT with the use of an immobilization device and an IGRT system were evaluated. This study showed variability in random translational errors for different regions in the anatomy of head and neck cancer patients due to rotational shifts happening inter and intrafraction. It also estimated rotational shift using stereoscopic projections and provided estimates of mean and standard deviation.^18^ The intrafractional systematic (Σ) and random (σ) rotational displacements of the spine in the upper neck found from repeat stereoscopic projections^18^ would yield a needed coverage range of ±5 degree along one axis, using the Van Herk formula of 2.5 Σ + 0.7 σ for drawing a range of displacements to encompass likely target shift.^19^

Most previous studies of rotational effects have considered the geometric center of the gross tumor volume as their axis of rotation. We evaluated an off‐axis rotation around the spine, as this is the more likely anatomic axis of rotation.^20^ In this study, the hypothesis was that forming a margin that would cover the space occupied by an oropharyngeal CTV with ±5‐degree rotation around the spine would spare more of the pharyngeal constrictors than would current practice using an isotropic expanded PTV. We have retrospectively evaluated 20 patients with head and neck cancer who completed VMAT in our institute for oropharyngeal carcinomas. PTVs were specified on the physician request form to be 0.3 cm and formed isotropically. A new PTV was designed considering ±5 degree rotation along the spinal axis for each patient. The overall objective of this study was to evaluate the difference in the overlap between PTV and the pharyngeal constrictor muscles when using the original PTV based on isotropic expansion compared to a new PTV that tracked rotational deviations.

## 2 MATERIALS AND METHODS

**Fig. 1:**
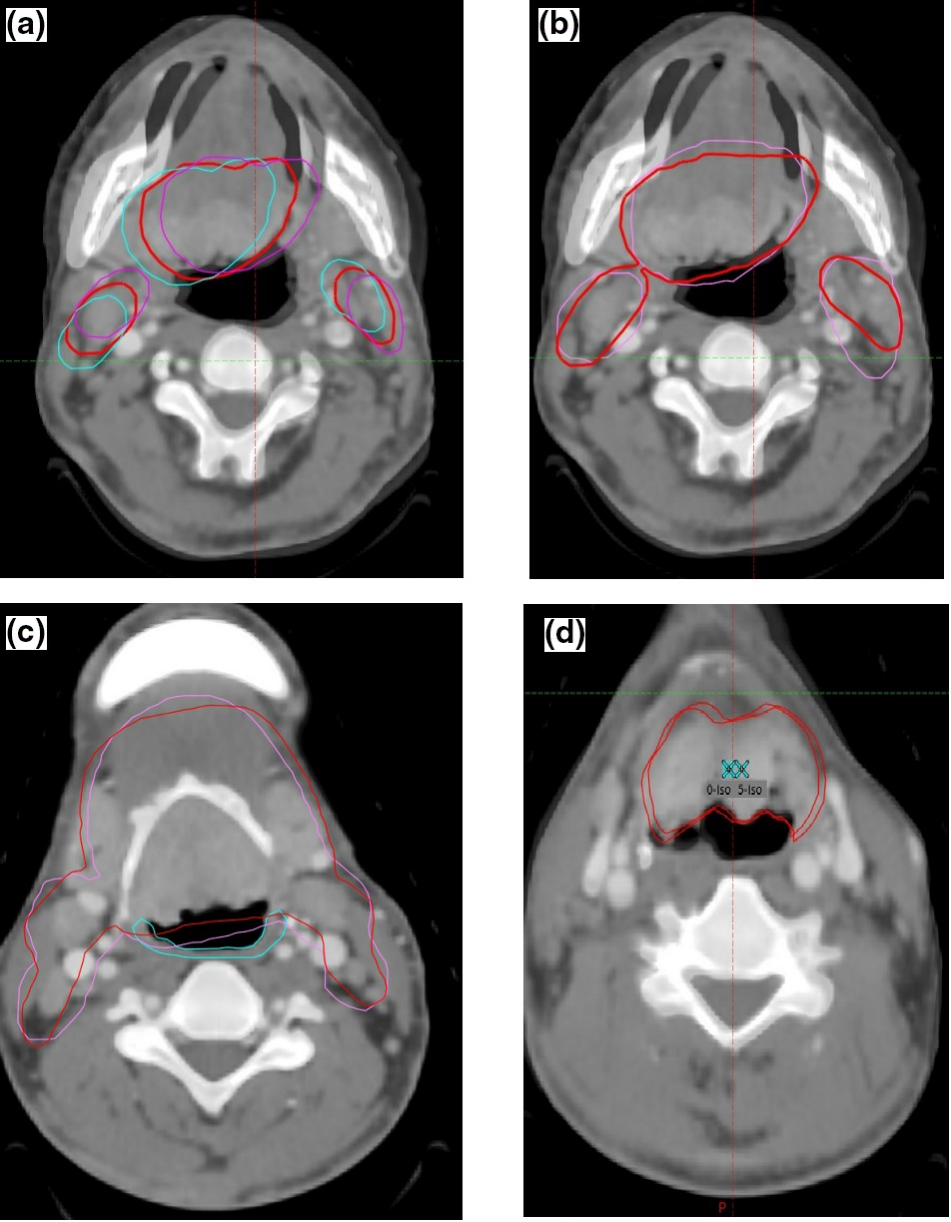
Demonstration of the rotational clinical target volumes (CTVs), standard planning target volume (PTV), new rotational PTV amd pharyngeal constrictor muscles (PCM) overlap. (a) CTV at zero degree rotation is in red. CTV at +5 degree rotation is depicted in purple and at −5 degree rotation is in blue. (b) The standard 3‐mm isotropic expansion PTV is shown in pink and the new rotational PTV which is a bolean of rotational CTVs is depicted in red. (c) The overlap between middle PCM (blue) and standard isotropic PTV (purple) versus the overlap between middle PCM and new rotational PTV (red). (d) Tumor isocenter at 0 degree is on the right and at 5‐degree rotation is on the left. The GTV and the 5‐degree rotation of the GTV are also ilustrated.

This study was approved by the IRB of our institute. All procedures followed were in accordance with the ethical standards of the responsible committee on human experimentation (institutional and national) and with the Helsinki Declaration of 1975, as revised in 2008. Since this was an IRB approved retrospective study reviewing treatment plans of patients who received radiation in our department, individual consent forms were not obtained. We retrospectively evaluated 20 VMAT plans of patients who received EBRT in our department for oropharyngeal cancer. The Eclipse treatment planning system, version 13.7 by Varian Medical systems, was used to generate the VMAT plans. Patients were immobilized using the Klarity S type head and shoulder mask. The treatment‐planning CT scan was acquired using 3‐mm slice scan with intravenous contrast. Since this is a retrospective study, all contours were done by the primary treatment team. The following contoured OARS were available: Brain stem, spinal cord, bilateral parotid, esophagus, trachea, brachial plexus, larynx, eye lens, optic nerves, and chiasm. Target contours included high‐risk CTV around the GTV, intermediate risk CTV and low‐risk CTV. For this study, we only considered the GTV with the high‐risk CTV expansion which included gross primary disease in addition to any involved node. A standard PTV was then generated using a uniform 3mm expansion from this CTV. A copy of the simulation CT scan was created and fused with the original image for rotational assessment. The image was rotated along the cervical spinal longitudinal axis. The available treatment planning system only allows rotations through isocenter in addition to translations. In order to perform an off axis rotation, a hypothetical point was placed on the copied image through the center of the cervical spinal canal on the transaxial plane containing the treatment isocenter at the level of C3. The copied image was then moved along the axial plane so that the point placed in the spinal canal coincided with the isocenter on the CT simulation image. The image was then rotated around the longitudinal axis ±5 degrees. The copied image was then moved back so that the rotation point returned to its original anatomic location on the simulation image which was at the center of the cervical spinal canal. This generates a new image of the CTV that represents its rotational shift along a longitudinal axis through the spinal canal, that is, off the isocenter.^21^ The rotational CTVs in 1‐ degree increments, including −5,‐4,‐3,‐2,‐1, 0, 1, 2, 3, 4, 5, were used to generate a new rotational PTV using the Boolean operation on Eclipse. Figure 1 illustrates the technique used. Figure 1(a) shows original CTV with ±5 degree rotation and Fig. 1(b) emphasizes on the standard isotropic PTV and the new rotational PTV.

In order to evaluate the overlap between new and standard PTV, Pharyngeal Constrictor Muscles (PCM) were separately contoured based on the contouring guideline published by Christianen et al.^22^ In summary, the superior PCM was contoured from the caudal tip of the pterygoid plate to the lower edge of C2, followed by the middle PCM, contoured from the upper edge of C3 to the lower edge of the hyoid bone. Finally, inferior PCM, which was defined only as the thyropharyngeal muscle component, was contoured from the lower edge of the hyoid bone to the lower edge of the arytenoid cartilage. The overlap volume between the standard PTV and each PCM was calculated using the Boolean operation and this was compared to the overlap volume between the new PTV and the PCMs as well [Fig. 1(c)].

The shift in the tumor center due to the off‐axis rotation about the spine was determined. In order to do this, a point was manually placed about the middle of the GTV, and the location of this point upon rotating the image about the spine was recorded [Fig. 1(d)].

In order to evaluate the effect of the rotational PTV on the mean PCM dose, one patient was randomly selected and the VMAT plan was generated using three dose levels, 70 Gy to GTV and positive nodes, 63 Gy to high‐risk volume and 54 Gy to bilateral elective cervical nodes. All PTV levels were designed using the same rotational technique. Based on the Nutting et al study,^23^ the mean dose to the volume of superior and middle PCM outside the high‐dose volume was set as a mandatory constraint and the expected mean dose was <50 Gy. 2 sets of plans were generated using the exact same dose constraints and optimization techniques. In the first plan, the standard isotropic expanded PTV was used and in the second plan, the rotational PTV was used instead. In order to calculate physician‐rated swallowing dysfunction in 6 months, the NTCP model discussed in Christianen et al paper was used.^24^ This model uses superior pharyngeal constrictor and supraglottic larynx mean dose for NTCP calculation.

JMP version 13 was used to analyze the data. Descriptive statistics and t‐test were used and *P* < 0.05 was considered as statistically significant.

## 3 RESULTS

With regard to the baseline tumor characteristics, 25% of patients had base of tongue lesion and 75% had tonsillar lesions. Sixty‐five percent of cases were human papilloma virus (HPV) positive. T2 was the most common T staging (35%) followed by T4 (30%), T1 (20%), and T3 (15%). Forty‐five percent of patients had N2 disease, 30% had N1, 15% had N0, and 10% had N3 disease. None of the patients had metastatic disease. All patients received bilateral elective cervical neck radiation.

Table 1 summarizes the overlap volume and percent change in the overlap using the new rotational PTV instead of the standard PTV. Average percent change for overlap with the superior, middle, and inferior PCMs are as followed: −37%, −59.4%, and −45.2%. Of note, the average percent change in the PTV volume was −19%.

**Table 1:** Difference in planning target volume (PTV) overlap with pharyngeal constrictor muscles for standard isotropic PTV vs new rotational PTV.

	Volume reduction^a^ average (range)	Percent change average (range)
Superior constrictor	0.61 (−0.28, 1.62)	−37% (−100%, 4.2%)
Middle constrictor	0.20 (0, 0.63)	−59.4% (−100%, −10.7%)
Inferior constrictor	0.07 (0‐0.91)	−45.2% (−93.7%, 0%)
PTV	35.2 (4.24, 89.84)	−19% (−31.4%, −5.81%)

^a^ Volume reduction calculated in cm^3^.

Since a 3‐mm CTV expansion is recommended to form the standard PTV, we evaluated this expansion to see if it covers the 5‐degree rotation used to form the rotational PTV. The smallest isotropic expansion that covers the new rotational PTV was between 3 and 5 mm for all patients, 65% covered by 3 mm expansion, 30% by 4 mm and 5% by 5 mm. That is, in one‐third of cases, a 3‐mm isotropic PTV expansion would not cover the CTV volume if the patient rotated through the spine by 5 degrees. The average tumor center shift due to off‐axis rotation along the spine was 0.49 cm with a range of 0.16–0.73 cm.

In order to evaluate the mean percent change using the new rotational PTV considering different tumor characteristics, BOT and tonsillar lesions were compared as well as T and N status. The new rotational PTV causes statistically significant reduction in the superior PCM overlap in the BOT lesions compared to tonsillar lesion, 57.8% vs 25.8%, *P* = 0.01, as well as middle PCM overlap, 73% vs 49%, *P* = 0.04 (Table 2). The new rotational PTV does not have a statistically significant different effect on tumors with different T and N staging (Table 3).

**Table 2:** Difference in planning target volume (PTV) overlap with pharyngeal constrictor muscles for standard isotropic PTV versus new rotational PTV by tumor site.

	Tonsil (mean percent)	BOT (mean percent)	*P*‐value
Superior constrictor	−25.8	−57.8	0.01*
Middle constrictor	−49	−73	0.04*
Inferior constrictor	−18	−20	0.47
PTV	−18	−20	0.46

* Statistically significant.

**Table 3:** Mean percentage change in planning target volume (PTV) and pharyngeal constrictor overlap for standard isotropic PTV vs new rotational PTV based on T and N class.

	T1	T2	T3	T4	p‐value	N0	N1	N2	N3	p‐value
Superior constrictor	−32.1	−32.6	−47.3	−40.3	0.87	−43.6	−41.7	−31.3	−39.6	0.88
Middle constrictor	−55.4	‐52.8	−77.2	−56.2	0.6	−57.6	−67.4	−57.9	−41.8	0.86
Inferior constrictor	−93.7	−50	N/A	−31.9	0.20	−50	−48.5	−62.1	0	0.57
PTV	−20.1	−18.04	−22.4	−17.9	0.1	−18.5	−23.1	−18.6	−9.5	0.21

**Graph 1:**
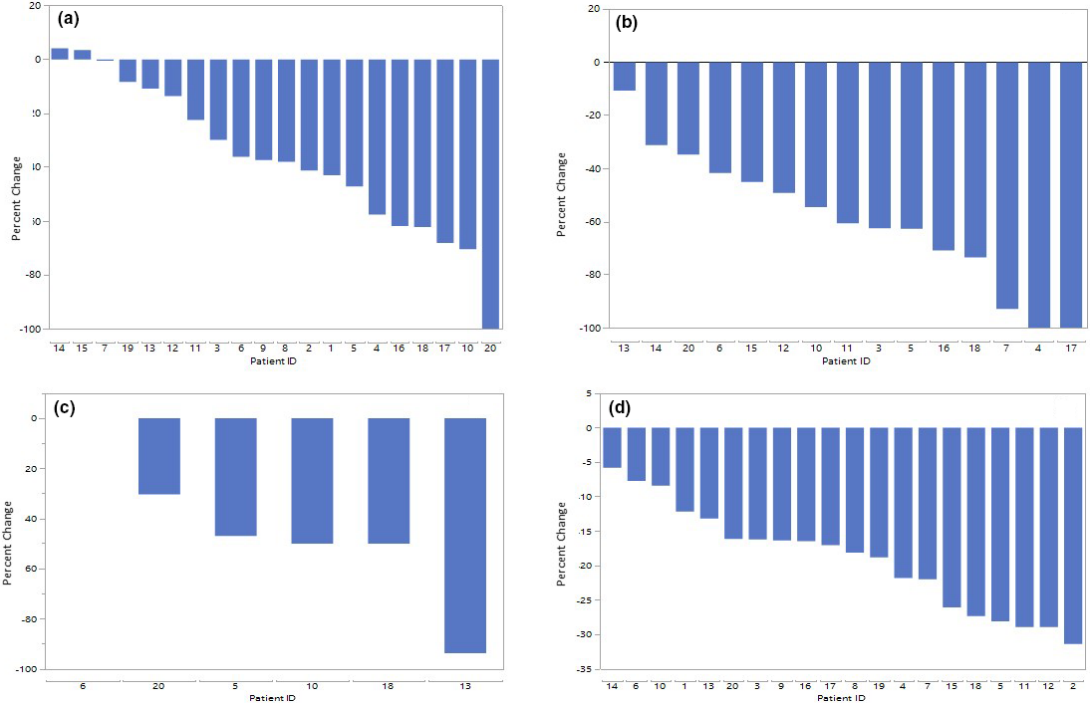
Percent change in each individual patient. (a) Percent change in the superior pharyngeal constrictor muscles (PCM) overlap using the new rotational planning target volume (PTV). (b) Percent change in the middle PCM overlap using the new rotational PTV (5 patients omited as there was no overlap between middle SCM and the standard and new rotational PTVs). (c) Percent change in the inferior PCM overlap using the new rotational PTV (14 patients omitted as there was no overlap between inferior PCM and the standard and new rotational PTVs). (d) Percent change in the new rotational PTV.

Graph 1 shows the mean percent change in each patient separately. There were five patients that did not have an overlap between middle PCM and standard or new rotational PTVs. In addition, 14 patients did not have an overlap between inferior PCM and standard or new rotational PTVs.

In order to evaluate the dosimetric data, patient 13 was randomly selected. Using the same dose constraints and optimization factors for both plans, the mean superior, middle and inferior PCM dose was 5138, 4638, and 4345 cGy in the standard plan vs 4574, 3818, and 3958 cGy. This resulted in significant reduction in the PCM dose while meeting all other dose constraints (Table 4). Based on the physician‐rated swallowing dysfunction in 6 months NTCP model, the dysphagia rate decreased from 21% to 14% in this patient.

**Table 4:** Dose to organs at risk and planning target volume (PTV) coverage summarized for 2 VMAT using standard and rotational PTV in 1 patient.

Organ at risk	Standard PTV plan (cGy)	Rotational PTV plan (cGy)
Mean PCM dose		
Superior	5138	4574
Middle	4638	3818
Inferior	4345	3958
Maximum spinal canal dose	4312	4229
Mean parotid dose	3152	2700
Mean submandibular gland dose	2984	2449
Maximum brainstem dose	3879	4026
95% of PTV receiving		
PTV70	6782	6878
PTV63	6232	6336
PTV54	5201	5279
100% of GTV70 receiving	6613	6655
Minimum GTV70 dose:	6646	6646

## 4 DISCUSSION

Per ICRU, PTV expansions have largely been built from recorded translational shifts only, neglecting rotational components.^10^ When rotations have been considered in PTV construction using sampling methods, the translational and rotational components have been treated as statistically independent by assuming a given and fixed rotation center.^25^ In this study, the hypothesis was to design the PTV based on ±5‐degree rotation instead of isotropic expansion of CTV. The rotation center was set in the center of the cervical spinal canal, which is the anatomic location for rotations in the head and neck region.^26^ Standard practice is to shift isocenter relative to the spine because its center is well detected on orthogonal KV projections, using the spinous process and borders of the vertebral body. Shifts in isocenter are well corrected but rotations are not well captured and require PTV to be designed. Although translational and some rotational errors (pitch and yaw) can be mitigated with KV orthogonal projections, residual roll error remains CBCT can allow better visualization of rolls but are not in practice done daily. Issues with daily CBCTs for head and neck treatments include time lag between imaging, adjustment, and treatment which contributes to treatment error, additional dose especially to the lens, and not qualifying for current insurance guidelines. A PTV that accounts for rotations, which is the residual source of error with available imaging and adjustment methods, would allow greater confidence in target irradiation than a PTV built from translational considerations only. This was the focus of this study to account for rotational errors. The result of our study revealed that the new rotational PTV is more effective in sparing the PCMs compared to the standard 3 mm isotropic expansion of the CTV. The average percent change in the PTV volume was −19%, and there were 37%, 59.4%, and 45.2% reductions in the superior, middle, and inferior constrictor overlap with the new rotational PTV compared to the standard PTV. Moreover in one third of cases a PTV with standard 3mm isotropic expansion would not cover the CTV in the rotated patient.

The percent change for superior and middle PCM overlap was more significant in cases with BOT lesions compared to tonsillar lesions. This might be due the anatomic location of the BOT lesions being closer to the PCMs. However, more cases are needed to confirm this difference as only 25% of patients had BOT lesions.

To date, no other study has used a similar technique to design rotational PTVs by using cervical spinal cord as the center of rotation. However, a study done by Arumugum et al used the isocenter that was located in the center of the target volume as the center of rotation and evaluated symmetrical rotational errors from −3 to +3 degrees.^27^ The impact of these errors on the dose to the standard PTV was then studied. In head and neck patients, the percentage difference in mean dose to PTV was around 0.2% to 3.2% with maximum percentage difference of up to −9.8% in D95 to PTV. This study emphasized the importance of correcting rotational errors to avoid overdosage of critical structures and underdosage of tumor volumes but did not evaluate overlap and dose difference to the pharyngeal constrictors.^27^ Another study by Samuels et al evaluated PTV elimination or dose reduction in patients with HPV positive oropharyngeal cancers. This study also considered the overlap between ipsilateral parotid gland and contralateral submandibular gland and the non‐expanded CTV. Considering Normal Tissue Complication Probability (NTCP), patients with more than 13% overlap with ipsilateral parotid gland and 22% overlap with contralateral submandibular gland showed a clinically significant improvement in NTCP.^28^

Studies have been done to evaluate the effect of reducing the dose to PCMs using different techniques. Van Kranen et al used three different isotropic expansions ranging from 5 to 0 mm. This study showed that margin reduction from 5 to 3 mm and then 0 mm resulted in organ‐at‐risk mean dose sparing of approximately 1 Gy/mm.^11^ This technique reduced the mean dose to pharyngeal constrictors from 54.3 to 52.1 Gy and then 49.4 Gy.^11^ A study done in Netherlands used a swallowing sparing IMRT technique. In this technique, additional objectives were used to spare swallowing organs including PCMs. This study showed that dose reduction was greatest in patients with neck irradiation, tumors located in the larynx, oropharynx, nasopharynx, or oral cavity, and <75% overlap between swallowing organs at risk and PTVs. This technique resulted in 6.1% reduction in grade 2–4 swallowing dysfunction.^29^

The main goal of this work was to introduce the concept of designing a PTV that accounts for potential rotational movements which can result in better tumor coverage and tissue sparing. We recognize that this work does not present a complete solution to the problem of incorporating rotational movement into PTV design, but instead shows the potential differences and benefit of developing a general solution. There are a few limitations in this study. Since determining the swept volume of the target rotating and translating is challenging, this study only focuses on rotation along the axial plane. Therefore, rotations in other planes and translations have not been considered. In addition, dose comparison is challenging due to the subjective nature of dose optimization and the biases inherent in optimizing against an obviously different PTV. However, our team managed to develop a similar dose constraint and optimization technique in one patient and showed a reduction in mean PCM dose. Our team is currently working on the dosimetric data and developing a technique to look at translations and rotations together.

## 5 CONCLUSION

This study proposed designing a new PTV based on rotational errors caused by cervical spinal rotation in patients with oropharyngeal cancer. This new rotational PTV resulted in significant reduction of the overlap volume between PCMs and PTVs without significant changes in the PTV volume. Further studies should be done in a larger population with additional evaluation of changes in dose distribution to OARs. A next step would be to evaluate the association between dose to PCMs and patient reported outcome including dysphagia in patients treated with the new rotational PTV technique.
